# SMG7 is a critical regulator of p53 stability and function in DNA damage stress response

**DOI:** 10.1038/celldisc.2015.42

**Published:** 2016-01-19

**Authors:** Hongwei Luo, Lauren Cowen, Guowu Yu, Wenguo Jiang, Yi Tang

**Affiliations:** 1 Center for Cell Biology and Cancer Research, Albany Medical College, Albany, NY, USA

**Keywords:** p53, SMG7, Mdm2, ATM, phosphorylation, DNA damage, transcription factor, cell cycle arrest

## Abstract

The p53 tumor suppressor functions as a transcription factor and plays a pivotal role in regulation of cellular response to DNA damage by activating various genes including those involved in cell cycle arrest. p53 stability is essential for its function during stress response; however, the molecular mechanism for DNA damage-induced stabilization of p53 is not fully understood. In our present study, we have identified SMG7 (suppressor with morphological defects in genitalia 7), also known as EST1C, as a novel p53-binding protein. SMG7 is an mRNA surveillance factor implicated in degradation of p53 mRNA-containing nonsense mutations, yet it is completely unknown whether SMG7 regulates p53 function. Here, we show that SMG7 has a crucial role in p53-mediated response to genotoxic stress by regulating p53 stability. Using somatic gene knockout, we found that deletion of *SMG7* abrogates DNA damage-induced p53 stabilization, although it exhibits minimal effect on the basal levels of p53. Importantly, loss of SMG7 impairs p53-mediated activation of *p21* and cell cycle arrest following DNA damage. Pharmacological inhibition of Mdm2, a major E3 ubiquitin ligase for p53, restored p53 stability in gamma-irradiated *SMG7*-deficient cells. Furthermore, SMG7 physically interacts with Mdm2 and promotes ATM-mediated inhibitory phosphorylation of Mdm2 following ionizing radiation. Therefore, our present data demonstrate that SMG7 is critical for p53 function in DNA damage response, and reveal the SMG7-mediated phosphorylation of Mdm2 as a previously unknown mechanism for p53 regulation.

## 1 Introduction

*p53* is a tumor suppressor gene that is inactivated by somatic mutations in the majority of human cancer [1]. The p53 protein, which primarily acts as a transcription factor, controls a gene network that modulates cellular response to diverse stresses such as DNA damage, activation of oncogenes, hypoxia, aberrant metabolism and defective ribosome biogenesis [2–5]. Described as the ‘guardian of the genome’, p53 has a crucial role in maintaining genome integrity by activating target genes to induce cell cycle arrest, DNA repair, senescence and apoptosis in response to varying degrees of genotoxic stress [3, 6]. These p53-dependent functions collectively prevent the proliferation of cells harboring unrepaired DNA lesions and contribute to p53-mediated tumor suppression [3].

As activation of p53 exerts strong inhibitory effects on cell growth and survival, the p53 protein and its transcriptional activity are normally maintained at low levels under normal conditions. Among numerous proteins involved in p53 regulation, Mdm2 is the major negative regulator controlling p53 levels and activities [7, 8]. The Mdm2 protein is encoded by the *Mdm2* oncogene, whose amplification has been frequently observed in soft tissue tumors, osteosarcomas and esophageal carcinomas [9]. Mdm2 contains an N-terminal p53-binding domain and a C-terminal RING domain that confers E3 ubiquitin ligase activity [7]. By physically interacting with p53, Mdm2 can repress p53-mediated transcriptional activation [10, 11] and induce p53 ubiquitination, which further leads to nuclear export of p53 and/or its degradation by the 26S proteasome [12–15]. The physiological significance of Mdm2-mediated inhibition of p53 has been demonstrated in animal studies under both normal and pathological settings. Deletion of the *Mdm2* gene in mice is embryonic lethal, and this lethality can be completely rescued by concomitant inactivation of p53 [16, 17], indicating that Mdm2 is required for the control of p53 functions during normal embryonic development. In tumor studies, mice engineered to overexpress Mdm2 exhibit accelerated spontaneous tumorigenesis associated with reduced p53 levels and activities [18, 19]. Taken together, literature has well-established Mdm2 as a critical regulator of p53 functions in normal cell and physiological contexts.

In response to DNA damage, the p53 protein is stabilized and activated to induce expression of various target genes involved in cell cycle arrest, senescence and apoptosis [6]. p53 stabilization, a key step in activating gene transcription, is mainly achieved through inhibition of Mdm2-mediated ubiquitination and degradation of p53. Early studies have shown that ATM (Ataxia-Telangiectasia Mutated), a member of the conserved PI3K-like protein kinase family and key signaling component in cellular response to DNA double strand breaks [20, 21], is required for p53 stabilization following ionizing radiation [22]. As activation of ATM induces p53 phosphorylation at the N-terminal sites Ser15 and Ser20, located in the Mdm2 binding domain of p53 [23–25], it was initially suggested that these modifications stabilize p53 by disruption of the interaction of p53 with Mdm2. However, this model of p53 stabilization is not supported by cell culture studies, which demonstrate that phosphorylation of p53 at these sites is dispensable for its stabilization [26, 27]. Animal studies also show that phosphorylation of Ser15 and Ser20 may modulate gene transactivation by p53 but only has a very mild effect on p53 stabilization after DNA damage [28–30], suggesting that additional mechanisms other than ATM-mediated phosphorylation of p53 must exist to regulate p53 stabilization.

Although DNA damage-induced ATM phosphorylation of Mdm2 was discovered over a decade ago [31], only recently has it been shown that this modification is critically involved in p53 stabilization. It was first reported that ATM phosphorylation of Mdm2 at Ser395 is induced by ionizing radiation and phosphorylation-mimic S395D mutant Mdm2 exhibits less potent degradation of p53 when expressed in cultured cells [32]. Several other ATM sites such as Ser386 and Ser429 were later identified [33], and the corresponding data indicate that ATM-mediated phosphorylation of Mdm2 at these sites near the RING domain inhibits Mdm2 oligomerization and E3 ligase activity [33, 34]. Recently, *in vivo* studies utilized mice bearing altered *Mdm2* alleles to show that ATM phosphorylation of Mdm2 at serine 395 is required for robust p53 stabilization and activation after DNA damage [35]. Importantly, mice expressing mutant Mdm2 S395A develop spontaneous lymphoma at a rate similar to *p53*-hemizygous animals, suggesting that Mdm2 Ser395 phosphorylation contributes to effective p53-mediated tumor suppression.

Despite recent progress, the molecular mechanisms of the p53 response to genotoxic stress remain to be fully elucidated. To gain insight into p53 regulation, we purified an endogenous p53-containing protein complex from gamma-irradiated cells and identified SMG7 as a previously unknown p53-binding protein. Using homologous recombination-mediated somatic gene knockout, we found that SMG7 is required for p53 stabilization and activation after DNA damage. Intriguingly, SMG7 physically binds Mdm2 and is crucial for the ATM-mediated phosphorylation of Mdm2 at several serine residues including Ser395 induced by ionizing radiation. Our studies demonstrate that SMG7 is critical for the p53 response to DNA damage by promoting Mdm2 phosphorylation.

## Results

### Affinity purification of an endogenous p53-containing protein complex

**Figure 1:**
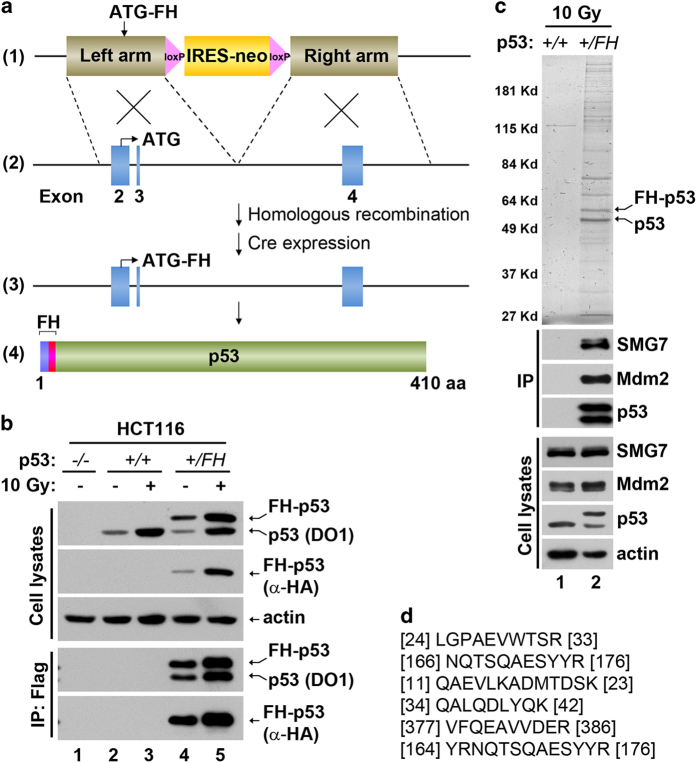
Identification of SMG7 as a p53-associated protein. (**a**) Scheme of AAV-mediated epitagging of *p53*: (1) AAV-*p53* targeting vector; (2) wild-type *p53* allele; (3) modified *p53* allele containing the Flag-HA cassette; (4) FH-tagged p53 polypeptide. See details of generation of HCT116 *p53*^*+/FH*^ cells in Materials and Methods. (**b**) Cell extracts from control or irradiated cells (10 Gy, 4 h) and the corresponding α-Flag immunoprecipitates were analyzed with western blot analysis using antibodies as indicated. (**c**) Silver staining of affinity-purified p53-containing protein complex from irradiated cells (upper panel). Cell extracts and the Flag/HA immunoprecipitates were analyzed with western blot analysis using antibodies as indicated. (**d**) Several representative SMG7 peptide sequences identified using mass spectrometric analysis. Numbers indicate the overlapping position of the peptides with SMG7 sequence (NP_963862).

Studies using affinity purification in combination with mass spectrometric analysis have led to the identification of a number of p53-binding proteins that regulate various aspects of p53 function [36–39]. As expression of wild-type p53 induces cell growth arrest and apoptosis, stable cell lines engineered to express exogenous p53 mutants that are incapable of these activities were utilized in the previous studies. To isolate p53-associated proteins under physiological conditions, we modified the *p53* gene using an AAV (adeno-associated virus)-based gene-targeting approach in HCT116 cells, a human colorectal cancer cell line that retains wild-type *p53* [40]. Through homologous recombination between the *p53* exon 2/3 region and the corresponding DNA sequence in the viral vector, an expression cassette encoding the Flag-hemagglutinin (HA) (FH) double tag was inserted in-frame immediately after the ATG start codon in one of the two *p53* alleles (Figure 1a). Clones containing targeted insertion were identified by PCR-based genotyping (Supplementary Figure S1A) and expression of the FH-p53 protein from the endogenous *p53* promoter was then achieved after Cre recombinase-mediated removal of the IRES-neo cassette. As expected, similar to the unmodified p53, FH-p53 was expressed and strongly induced in the heterozygous HCT116 *p53*^*+/FH*^ cells after ionizing radiation (Figure 1b, lane 4 versus lane 5). Importantly, Flag-mediated immunoprecipitation (IP) pulled down both tagged and untagged p53 because of the formation of p53 tetramers, which allows efficient capture of proteins associated with p53.

In search for novel p53-interacting proteins that potentially regulate its function in cellular response to DNA damage, we prepared whole-cell extracts from parental and *p53*^*+/FH*^ HCT116 cells following ionizing radiation and performed tandem affinity purification using agarose beads conjugated with α-Flag and α-HA antibodies. As shown in Figure 1c, the purified materials from HCT116 *p53*^*+/FH*^ cells were highly specific to p53, as nearly all the protein bands were not observed from the control cells (upper panel). Using mass spectrometric analysis, we identified a number of proteins known to interact with p53 either directly or indirectly (Supplementary Figure S1B), which validated our approach. Interestingly, SMG7 was identified as a previously unknown p53-interacting protein, whose unique matching peptide sequences are shown in Figure 1d. The high spectral abundance factor value suggests that SMG7 is likely a major p53-associated protein in cells following DNA damage (Supplementary Figure S1B). To corroborate the findings from mass spectrometry, we performed western blot analysis using an α-SMG7 antibody and demonstrated that SMG7 was indeed present in the p53-specific immunoprecipitated materials (Figure 1c, lower panels).

### SMG7 is a bona fide p53-binding protein

**Figure 2:**
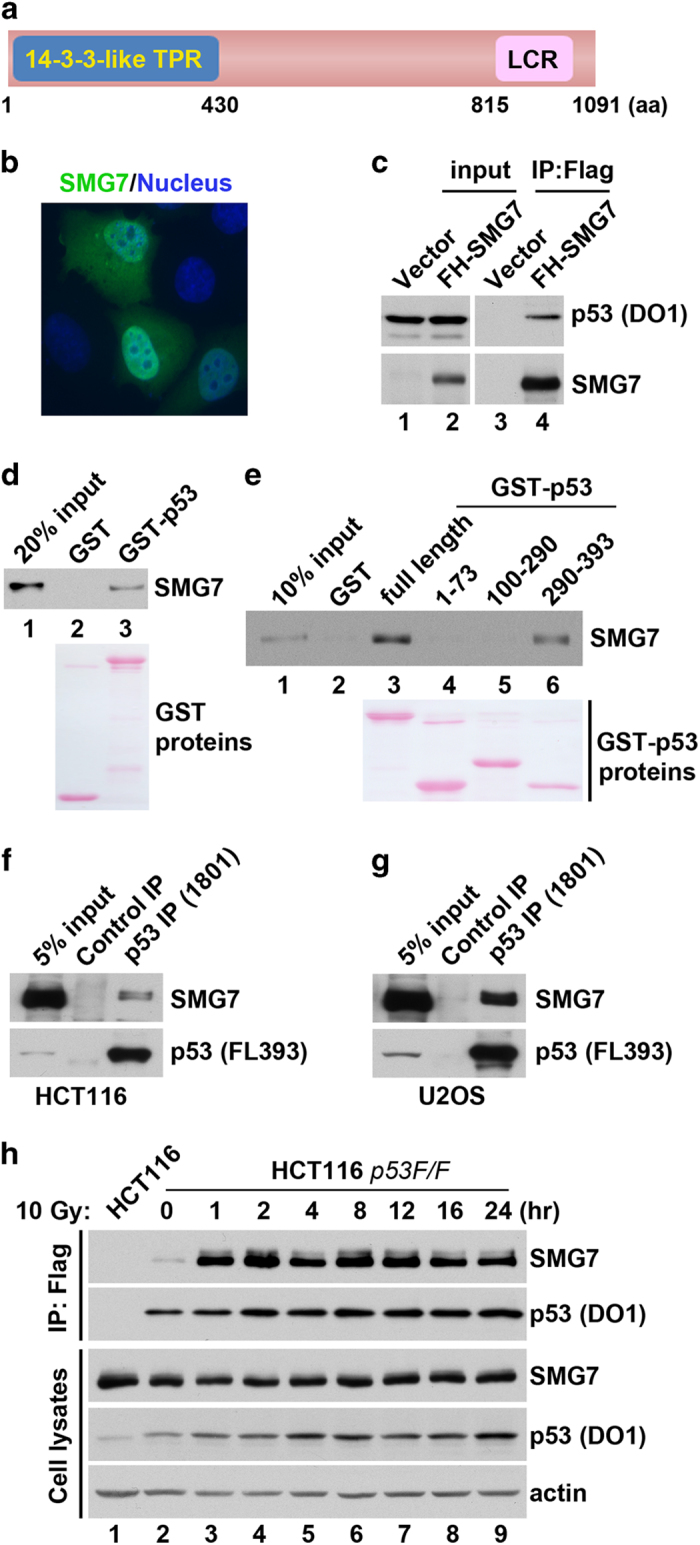
*In vitro* and *in vivo* interactions between SMG7 and p53. (**a**) Scheme of human SMG7 protein containing the N-terminal 14-3-3-like Tetratricopeptide repeat (TPR) and C-terminal low complex region. (**b**) Immunofluorescence microscopy of H1299 cells transiently expressing FH-SMG7 stained with α-HA antibody (green) and 4,6-diamidino-2-phenylindole (DAPI; blue). (**c**) Cell extracts from H1299 cells transiently expressing p53 with or without FH-SMG7 and the α-Flag immunoprecipitates were analyzed using western blot using α-SMG7 and α-p53 antibodies. (**d**, **e**) SMG7 binding to p53 *in vitro*. GST or GST-p53 fusion proteins (full length or various fragments) were used in pulldown assays with purified FH-SMG7 (**d**) or *in vitro* translated FH-SMG7 (**e**). FH-SMG7 was detected by western blot and GST proteins stained with Ponceau S. (**f**, **g**) Endogenous interaction of SMG7 with p53 in HCT116 (**f**) and U2OS cells (**g**). Cell extracts and the α-normal mouse IgG or α-p53 immunoprecipitates were analyzed with western blot analysis using α-SMG7 and α-p53 antibodies. (**h**) Cell extracts from control or irradiated cells (at various time points) and the α-Flag immunoprecipitates were analyzed with western blot analysis using antibodies as indicated.

*SMG7* was first identified as a genetic suppressor that regulates degradation of mRNA containing premature stop codons in *Caenorhabditis elegans* [41]. Recent studies have shown that SMG7 may act as an adaptor protein in the nonsense-mediated mRNA decay pathway [42, 43] and is involved in regulation of premature stop codon-containing p53 mRNA [44]. Interestingly, the SMG7 N-terminal region is structurally conserved with the 14-3-3 adaptor proteins (Figure 2a) [42], which can bind p53 and modulate its function [45–47], suggesting that SMG7 may have a role in the regulation of p53 function. To investigate the interaction between SMG7 and p53, we first examined the cellular localization of SMG7. Using immunofluorescence, we found that SMG7 is localized in both the nucleus and cytoplasm when transiently expressed in human lung cancer H1299 cells (Figure 2b). To validate the interaction of SMG7 with p53 in cells, we performed the IP assays. As shown in Figure 2c, p53 was only detected in the α-Flag immunoprecipitates from cells expressing p53 and F-SMG7 but not from cells expressing p53 alone (lanes 3 versus 4), indicating that p53 was co-immunoprecipitated with SMG7. Similarly, it was observed that SMG7 could be co-immunoprecipitated with F-p53, when they were transiently expressed in human embryonic kidney 293 cells (Supplementary Figure S2A). As previous studies showed that p53 Ser378 phosphorylation mediates binding to 14-3-3 proteins [48, 49], we tested whether this modification is required for p53 interaction with SMG7. Similar to wild-type p53, mutant p53 with alanine, aspartic or glutamic acid substitution at Ser378 exhibited a similar capacity to interact with p53 (Supplementary Figure S2B), suggesting that differences exist in the p53-binding property between SMG7 and 14-3-3 proteins.

To further characterize the interaction between SMG7 and p53, we performed *in vitro* binding assays using purified recombinant proteins. As shown in Figure 2d, SMG7 was pulled down by immobilized GST-p53 but not GST proteins, indicating that SMG7 can bind p53 directly and does not require additional binding partners. Moreover, using various GST-p53 fragments, we determined that SMG7 binds to the 290-393 C-terminal domain of p53 but not the N-terminal transactivation domain or the middle DNA-binding domain (Figure 2e, lanes 4–6). To examine the endogenous interaction between SMG7 and p53, we carried out co-IP using whole-cell extracts from HCT116 cells. As shown in Figure 2f, SMG7 was immunoprecipitated using α-p53 but not a control antibody, indicating that SMG7 binds p53 in HCT116 cells under normal conditions. When cell extracts from the human osteosarcoma U2OS cells were used in the same assays, we obtained similar results (Figure 2g). These data demonstrate that SMG7 is a bona fide p53-binding protein.

To test whether the interaction between SMG7 and p53 is regulated by DNA damage, we performed IP assays using HCT116 *p53*^*F/F*^ cells (a generous gift from Dr Waldman and Dr Kim) that express F-p53 from the endogenous promoter of *p53* [40]. As shown in Figure 2h, SMG7 was only detected in the α-Flag immunoprecipitates from the *p53*^*F/F*^ cells but not control HCT116 cells (lane 1 versus lane 2), which confirms the basal SMG7-p53 binding *in vivo*. Interestingly, the amounts of SMG7 in the α-Flag-immunoprecipitated materials were dramatically increased following ionizing radiation (lanes 3–9 versus lane 2), revealing the formation of a robust SMG7-p53 protein complex following DNA damage. Furthermore, similar observations were made when cells treated with Doxorubicin were used in the co-IP experiments (Supplementary Figure S2C). Notably, treatment with Nutlin [50], an inhibitor of Mdm2, strongly stabilized p53 but had no effect on its interaction with SMG7 (Supplementary Figure S2C, lane 2 versus lane 9), suggesting that the DNA damage-enhanced interaction between SMG7 and p53 is not simply a result of the increased p53 levels.

### SMG7 regulates p53 stability under DNA damage condition

**Figure 3:**
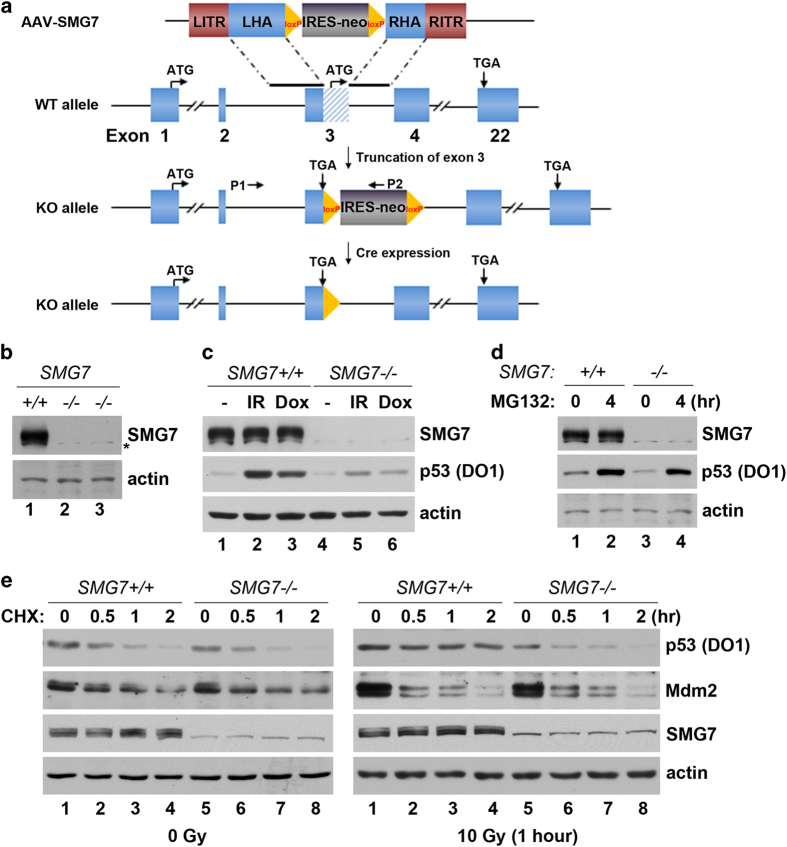
Knockout of *SMG7* abrogates DNA damage-induced p53 stabilization. (**a**) Scheme of AAV-mediated knockout of *SMG7*: shown from top to bottom are AAV-*SMG7* targeting vector, wild-type *SMG7* allele, knockout *SMG7* allele containing the IRES-neo cassette and knockout *SMG7* allele after removal of the IRES-neo cassette. See details of generation of HCT116 *SMG7*^*−/−*^ cells in Materials and Methods. (**b**) Western blot analysis of SMG7 in HCT116 and two *SMG7*^*−/−*^ clones. Asterisk indicates a nonspecific band. (**c**) Cell extracts from control, irradiated (10 Gy, 4 h) and Doxorubicin-treated cells (200 ng ml^−1^, 4 h) were analyzed using western blot analysis with α-SMG7, α-p53 and α-actin antibodies. (**d**) Cell extracts from control and MG132-treated (20 μM, 4 h) cells were analyzed using western blot analysis as in **c**. (**e**) Control or irradiated cells were harvested at various time points after Cycloheximide (CHX, 50 μg ml^−1^) treatment and cell extracts were analyzed using western blot analysis with α-SMG7, α-p53, α-Mdm2 and α-actin antibodies.

To study the function of SMG7, we sought to knockout *SMG7* in HCT116 cells using AAV-mediated gene targeting. Gene analysis suggests that the SMG7 mRNA is likely translated from two alternative start codons located in exons 1 and 3, respectively; therefore, our targeting strategy was designed to achieve simultaneous removal of the start codon and insertion of a stop codon in-frame in exon 3 following homologous recombination via the AAV-SMG7 targeting vector and the corresponding genome region (Figure 3a). To this end, HCT116 cells were infected with the viruses made using AAV-SMG7 vector, followed by selection with the antibiotic G418 and PCR-based screening. Remarkably, we observed very high targeting efficiency from the screening, as nearly 70% of G418-resistant clones showed integration of the neo cassette into the correct *SMG7* genome site (Supplementary Figure S3A). After the second round of gene targeting, we obtained several clones with both *SMG7* alleles inactivated, as demonstrated by PCR genotyping using primers flanking exon 3 (Supplementary Figure S3B). As expected, the *SMG7* knockout cells express similar levels of SMG7 mRNA compared with wild-type cells (Supplementary Figure S3C); however, they fail to produce the SMG7 proteins (Figure 3b).

**Figure 4:**
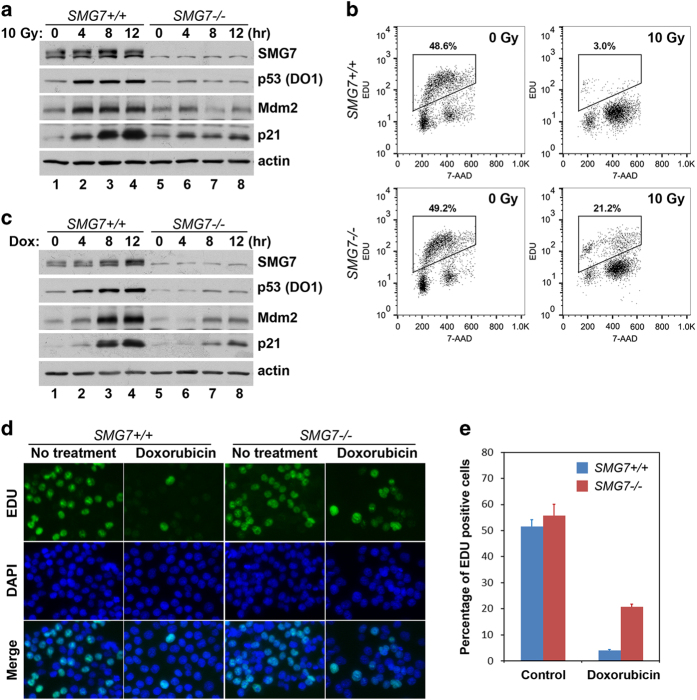
SMG7 regulates *p21* activation and cell cycle arrest upon DNA damage. (**a**) Total cell extracts from control or irradiated cells (at indicated time points) were analyzed with western blot analysis using antibodies against SMG7, p53, Mdm2, p21 and actin. (**b**) Control or irradiated cells (16 h) were treated with 5-ethynyl-2′-deoxyuridine (EDU) (10 μM) for 1 h and then processed for EDU and 7-AAD staining, followed by flow cytometry analysis. (**c**) Total cell extracts from control or Doxorubicin-treated cell (200 ng ml^−1^ for various time points) were analyzed by western blot as in **a**. (**d**) Control or Doxorubicin-treated (200 ng ml^−1^, 16 h) cells were fed with EDU (10 μM) for 1 h and then processed for EDU (green) and DAPI (blue) staining, followed by fluorescence microscopy. (**e**) Quantitation of EDU-positive cells from **d**. Error bars indicate s.d. calculated from three independent experiments.

To investigate the role of SMG7 in regulation of p53, we sought to determine whether p53 protein stability is affected by *SMG7* knockout. First, we examined p53 mRNA, as SMG7 has been shown to be involved in degradation of p53 mRNA containing nonsense mutations [44]. Using semiquantitative RT-PCR, we found that levels of p53 mRNA were not significantly altered in *SMG7*^*−/−*^ cells (Supplementary Figure S3D). Consistent with this observation, western blot analysis showed that the steady-state levels of p53 protein were similar between wild-type and *SMG7*^*−/−*^ cells (Figure 3c, lane 1 versus lane 4). Interestingly, induction of p53 4 h following ionizing radiation was strongly reduced in the absence of SMG7 (lane 2 versus lane 5) and similar observation was made when cells were treated with DNA damage-inducing agent Doxorubicin (lane 3 versus lane 6). It is worth noting that p53 stabilization was impaired in *SMG7*^*−/−*^ cells even after DNA damage treatment for extended time points (Figure 4a and b and Supplementary Figure S3E and F). Importantly, treatment with the proteasome inhibitor MG132 stabilized p53 in both wild-type and *SMG7*^*−/−*^ cells (Figure 3d, lanes 2 and 4), indicating that p53 is still subjected to proteasome-dependent degradation regardless of the *SMG7* status. Thus, these results suggest that SMG7 is required to stabilize p53 following DNA damage but not to maintain the basal level of p53.

To corroborate this finding, we assessed p53 degradation in *SMG7*^*−/−*^ cells before and after ionizing radiation. To this end, cells were treated with the protein translation inhibitor Cycloheximide and the levels of p53 were assayed at various time points using western blot analysis. As shown in Figure 3e, the amounts of p53 remaining in the cells after inhibition of protein synthesis at each time point similarly decreased in both wild-type and *SMG7*^*−/−*^ cells (left panel, lanes 2–4 versus lanes 6–8), indicating that p53 degradation is normal in unstressed *SMG7*^*−/−*^ cells. This is also consistent with the finding that knockout of *SMG7* appears to not significantly affect the basal levels of p53. However, upon ionizing radiation-induced DNA damage, p53 levels largely remained constant for up to 2 h in wild-type cells but steadily decreased in *SMG7*^*−/−*^ cells (Figure 3e, right panel, lanes 1–4 versus lanes 5–8). Quantitative analysis showed a dramatic difference in the p53 half-life between wild-type cells (~120 min) and *SMG7* knockout cells (more than 30 min; Supplementary Figure S3G). These data reveal a critical role for SMG7 in stabilizing p53 under DNA damage condition. We also examined Mdm2, the major E3 ligase for p53, under these conditions and found that the levels of Mdm2 were not significantly affected by loss of SMG7 before or after irradiation (Figure 3e), suggesting that SMG7 may promote p53 stability via mechanisms other than regulation of Mdm2 levels.

### SMG7 regulates p53-mediated DNA damage response

As p53 stabilization upon DNA damage is crucial to activate its target genes, our finding that p53 is not stabilized in the irradiated *SMG7*^*−/−*^ cells led us to further examine whether SMG7 regulates gene activation by p53. To this end, cells exposed to 10 Gy of ionizing radiation were harvested at various time points and the total cell extracts were analyzed by western blot analysis. As shown in Figure 4a, p53 was rapidly stabilized in wild-type cells but not in *SMG7*^*−/−*^ cells following ionizing radiation. Importantly, induction of p21 (a major p53-dependent target involved in cell cycle arrest) [51], although robust in wild-type cells, was nearly abolished in *SMG7*^*−/−*^ cells (Figure 4a). We also found that induction of Mdm2, a p53 transcriptional target, was markedly inhibited in the absence of SMG7. To directly examine transcriptional activation, we performed quantitative PCR to show that the mRNA levels of *p21* and *Mdm2* after irradiation were indeed significantly reduced in *SMG7*^*−/−*^ cells (Supplementary Figure S4A), indicating that SMG7 is required for p53 activation following ionizing radiation. It was previously shown that activation of p53 and subsequent induction of p21 cause cell cycle arrest at G1 by inhibiting the G1–S transition [52, 53]. To test whether SMG7 has a role in the p53-mediated cell cycle control, we monitored entry of cells into the S phase by EDU incorporation (which labels cells undergoing DNA synthesis) and flow cytometry. As shown in Figure 4b, the percentage of wild-type cells in S phase was dramatically reduced from 48.6 to 3.0% following ionizing radiation. However, the irradiated *SMG7*^*−/−*^ cells still showed a substantial EDU-positive fraction (21.2%), indicating that SMG7 has a critical role in the inhibition of the G1–S transition following DNA damage.

To corroborate these findings, we treated cells with the DNA damage-inducing agent Doxorubicin for various time points and analyzed p21 and Mdm2 expression and cell growth inhibition. Notably, western blot analysis and quantitative PCR showed that p53 stabilization and induction of p21 and Mdm2 (at both protein and mRNA levels) were significantly reduced in *SMG7*^*−/−*^ cells compared with wild-type cells after treatment with Doxorubicin (Figure 4c and Supplementary Figure S4B). Moreover, we found that both wild-type and *SMG7*^*−/−*^ cells had similar fractions of EDU-positive cells before treatment (between 50 and 60%) when measured via fluorescence microscopy (Figure 4d and e). Remarkably, less than 5% of wild-type cells were labeled by EDU after Doxorubicin treatment, whereas more than 20% of *SMG7*^*−/−*^ cells showed EDU-positive staining, indicating that SMG7 is necessary for the robust cell growth inhibition induced by Doxorubicin. When a second independent *SMG7*^*−/−*^ cell line was examined for p53 stabilization, activation of *p21* and cell cycle progression, we obtained similar results (Supplementary Figure S4C and D). These data demonstrate that SMG7 is required to block cell-cycle progression following DNA damage.

Early studies show that, following ionizing radiation, wild-type HCT116 cells are able to sustain a long-term cell cycle arrest, whereas *p53*- or *p21*-deficient cells continue to grow and eventually undergo apoptosis [54, 55]. To investigate the role of SMG7 in long-term DNA damage response, we performed colony formation assay and found that there was little difference between wild-type and *SMG7*^*−/−*^ cells (Supplementary Figure S4E). As both cell growth arrest and death prevent colony formation, we tested whether loss of *SMG7* sensitizes cells to apoptosis in response to DNA damage. By Hoechest 33258 staining, we found that *SMG7*^*−/−*^ cells showed significantly higher fractions of nuclei with condensed chromatin or nuclear fragmentation (characteristics of apoptotic cells) than wild-type cells after treatment with Doxorubicin or irradiation for 24 or 48 h (Supplementary Figure S4F and G), indicating that SMG7 protects cells from apoptosis after prolonged DNA damage. Given the critical role of SMG7 in p53 stabilization and activation, these data are consistent with previous studies showing that *p53*^*−/−*^ HCT116 cells are highly sensitive to DNA damage-induced apoptosis [55, 56].

### SMG7 binds Mdm2 *in vitro* and *in vivo*

**Figure 5:**
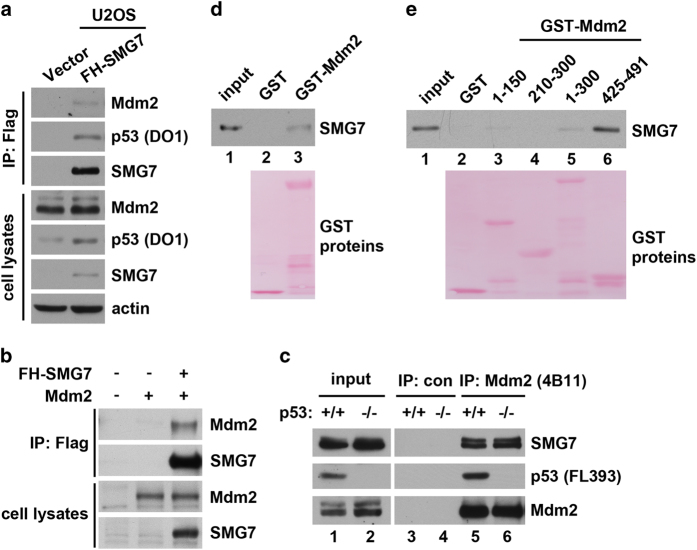
SMG7 physically interacts with Mdm2. (**a**) Cell extracts and the α-Flag immunoprecipitates were analyzed with western blot analysis using antibodies as indicated. (**b**) Cell extracts from H1299 cells transiently expressing Mdm2 with or without FH-SMG7 and the α-Flag immunoprecipitates were analyzed with western blot analysis using α-SMG7 and α-Mdm2 antibodies. (**c**) Endogenous interaction of SMG7 with Mdm2. Cell extracts and the α-normal mouse IgG or α-Mdm2 immunoprecipitates were analyzed with western blot analysis using antibodies as indicated. (**d**, **e**) SMG7 binding to Mdm2 *in vitro*. GST, full-length GST-Mdm2 (**d**) or various GST-Mdm2 fragments (**e**) were used in pulldown assays with purified FH-SMG7. GST proteins were stained by Ponceau S and FH-SMG7 was detected with western blot analysis.

Our results thus far have demonstrated that SMG7 has a critical role in stabilizing and activating p53 under DNA damage conditions. As inhibition of Mdm2, the major p53-negative regulator, is essential for p53 stabilization and activation, we reasoned that SMG7 might regulate Mdm2. To assess this possibility, we first tested whether SMG7 can interact with Mdm2 in cells. As shown in Figure 5a, we generated stable U2OS cell lines that express exogenous N-terminal Flag-tagged SMG7. By co-IP assay, we demonstrated that SMG7 interacts with p53 in the cells, as expected. Importantly, Mdm2 was also detected in the α-Flag immunoprecipitates from the stable cells expressing Flag-SMG7 but not control U2OS cells (Figure 5a), indicating that Mdm2 interacts with SMG7 in cells. Notably, Mdm2 and SMG7 were co-immunoprecipitated when they were transiently expressed in the p53-null H1299 cells (Figure 5b), indicating that p53 is not required for the interaction of SMG7 with Mdm2.

We also examined the *in vivo* interaction between endogenous SMG7 and Mdm2 proteins. As shown in Figure 5c, SMG7 was detected in the α-Mdm2 immunoprecipitates but not the immunoprecipitated material obtained with control antibodies from HCT116 cells (lane 3 versus lane 5). Similar observations were made with HCT116 *p53*^*−/−*^ cells (lane 4 versus lane 6). Using purified proteins, we carried out *in vitro* binding experiments to demonstrate that SMG7 can directly bind Mdm2 (Figure 5d, lane 2 versus lane 3). Moreover, using different GST-Mdm2 fragments, we found that SMG7 primarily interacts with the C-terminal RING domain-containing region (425–491; Figure 5e, lane 6) but not the middle acid domain (210–300; lane 4), and there appeared to be a weak interaction between SMG7 and the p53-binding domain (1–150) of Mdm2 (lanes 3 and 5). These results demonstrate that SMG7 binds Mdm2 *in vitro* and *in vivo* independently of p53.

### SMG7 regulates p53 stabilization in an Mdm2-dependent manner

**Figure 6:**
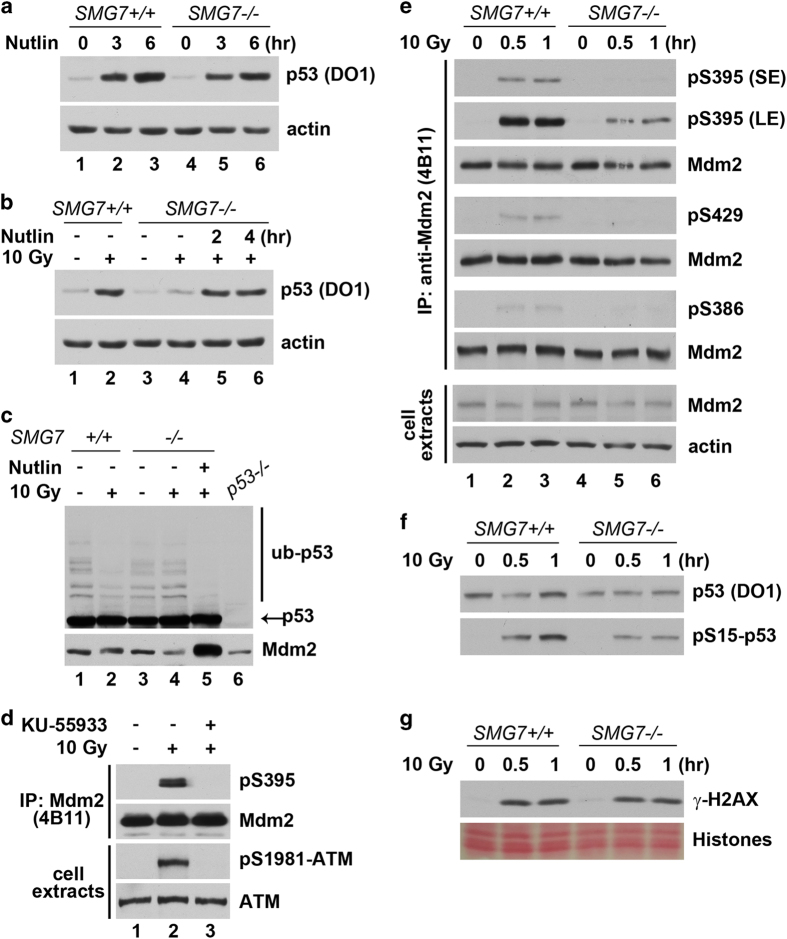
SMG7 is required for ATM-mediated phosphorylation of Mdm2. (**a**) Cell extracts from control or Nutlin (10 μM)-treated cells were analyzed with western blot analysis using α-p53 and α-actin antibodies. (**b**) Control or irradiated cells (10 Gy, 1 h) were harvested after Nutlin (10 μM) treatment and cell extracts were analyzed with western blot analysis as in **a**. (**c**) Cells treated as in **b** were lysed in 1× Laemmli SDS-PAGE sample buffer with 10 mM Iodoacetamide and immediately boiled. The whole-cell lysates with normalized amounts of unmodified p53 proteins were analyzed using western blot analysis with α-p53 and α-Mdm2 antibodies. (**d**) Cells were treated with MG132 (20 μM) for 4 h, and then incubated with or without KU-55933 (10 μM) for 1 h, followed by irrdiation (IR) (10 Gy, 1 h). Total cell extracts and the α-Mdm2 (4B11) immunoprecipitates were analyzed using western blot analysis with α-pS395-Mdm2, α-Mdm2, α-ATM and α-pS1981-ATM antibodies. (**e**) Cells were treated as in **d** and harvested for 0.5 or 1 h after IR. Cell extracts and the α-Mdm2 (4B11) immunoprecipitates were analyzed using western blot analysis with antibodies as indicated. (**f**, **g**) Cells were treated as in **e**, and the total extracts were analyzed using western blot analysis with α-p53 (DO-1) and α-S15-p53 antibodies (**f**), and α-γH2AX antibody (**g**). Total histone levels were shown by Ponceau S staining.

The physical interaction between SMG7 and Mdm2 suggests that SMG7 may directly regulate Mdm2 to stabilize p53 following DNA damage. To delineate the connection between SMG7 and Mdm2, we first tested whether inhibition of Mdm2 could stabilize p53 in *SMG7*^*−/−*^ cells. To this end, we exploited the specific Mdm2 inhibitor Nutlin, a small compound that can stabilize p53 by inhibiting the binding between Mdm2 and p53. By western blot analysis, we found that the p53 levels were similarly increased in wild-type and *SMG7*^*−/−*^ cells following treatment with Nutlin (Figure 6a). Although ionizing radiation failed to stabilize p53 in the absence of SMG7 (Figure 6b, lane 2 versus lane 4), treatment with Nutlin completely rescued p53 stabilization in the irradiated *SMG7*^*−/−*^ cells (lane 4 versus lanes 5–6). These results demonstrate that p53 is still subjected to Mdm2-mediated degradation in *SMG7* knockout cells under DNA damage condition, indicating that SMG7 has an inhibitory effect on Mdm2 degradation of p53.

To corroborate these findings, we examined Mdm2-mediated ubiquitination of the endogenous p53 protein. As shown in Figure 6c, p53 and its ubiquitinated forms were readily detected using western blot analysis in unstressed wild-type and *SMG7*^*−/−*^ cells (lane 6 versus lanes 1 and 3). Treatment with ionizing radiation significantly reduced p53 ubiquitination in wild-type cells but had no such effect in *SMG7*^*−/−*^ cells (lane 2 versus lane 4). However, treatment with Nutlin completely abolished p53 ubiquitination in the irradiated *SMG7*^*−/−*^ cells (lane 4 versus lane 5), suggesting that SMG7 likely stabilizes p53 via inhibition of Mdm2-mediated ubiquitination. Given that SMG7 can bind both Mdm2 and p53, and the interaction between SMG7 and p53 is highly induced following DNA damage, we attempted to test whether *SMG7* regulates the interaction between p53 and Mdm2. However, co-IP assays showed no significant difference in p53-Mdm2 binding between wild-type and *SMG7*^*−/−*^ cells before or after ionizing radiation (Supplementary Figure S5A). Thus, upon DNA damage SMG7 may stabilize p53 by inhibiting Mdm2 via other means.

### SMG7 is required for ATM phosphorylation of Mdm2

To gain insight into the molecular mechanism of SMG7-mediated p53 stabilization, we sought to examine whether SMG7 regulates ATM phosphorylation of Mdm2 in response to DNA damage. Several lines of evidence from this study and others support this hypothesis: (1) SMG7 physically interacts with Mdm2 *in vitro* and *in vivo*; (2) SMG7 enhances p53 stability after DNA damage; (3) ATM-mediated phosphorylation of Mdm2 (for example, Ser395) is a rapid response to DNA damage and is critical for p53 stabilization. To detect Ser395 phosphorylation of the endogenous Mdm2, we obtained a commercial antibody that was developed using phospho-peptide corresponding to the amino-acid residues surrounding Ser395 of human Mdm2. As it has not been reported in the literature that this antibody can be used in the detection of Ser395-phosphorylated Mdm2, we first tested its specificity. To this end, the recombinant Mdm2 proteins were *in vitro* phosphorylated by purified ATM and examined by western blot analysis. As shown in the Supplementary Figure S5B, this antibody recognized wild-type Mdm2 but not the Mdm2 S395A mutant after incubation with ATM. Moreover, the antibodies only reacted with wild-type, not the S395A mutant, Mdm2 when they were transiently expressed in cells exposed to ionizing radiation (Supplementary Figure S5C). These data confirm the antibody specificity as it only recognizes Mdm2 when it is phosphorylated at Ser395.

To assess Mdm2 Ser395 phosphorylation *in vivo*, we utilized this antibody to measure the levels of Ser395-phosphorylated Mdm2 in cells before and after DNA damage. The Mdm2 proteins were immunoprecipitated with a mouse monoclonal α-Mdm2 antibody (4B11) from cell extracts of untreated and irradiated HCT116 cells and examined by western blot analysis using the phosphorylation-specific antibody. As shown in Figure 6d, the level of Ser395-phosphorylated Mdm2 was undetectable in untreated cells but highly induced 1 h after ionizing radiation (lane 1 versus lane 2). Notably, treatment with the ATM-specific inhibitor KU-55933 before ionizing radiation completely blocked Mdm2 Ser395 phosphorylation (lane 2 versus lane 3), confirming that ATM is indeed the major kinase for this modification *in vivo*.

To elucidate the role of SMG7 in ATM-mediated Mdm2 phosphorylation, we carried out IP–western blot analysis as described above to measure the levels of Ser395-phosphorylated Mdm2 in wild-type and *SMG7*^*−/−*^ cells. As expected, ionizing radiation resulted in a rapid (0.5 h) induction of Mdm2 Ser395 phosphorylation in wild-type cells (Figure 6e, upper pS395 panels, lane 2 versus lane 1). However, ionizing radiation-induced Ser395 phosphorylation of Mdm2 was remarkably reduced in *SMG7*^*−/−*^ cells (lanes 2 and 3 versus lanes 5 and 6), indicating that SMG7 is crucial for ATM phosphorylation of Mdm2 at Ser395 *in vivo*. Furthermore, we also tested whether SMG7 regulates Mdm2 phosphorylation at two other ATM sites Ser386 and Ser429 using phosphorylation site-specific antibodies (generous gifts from Dr Jiandong Chen). As shown in Figure 6e, *SMG7* knockout significantly reduced ionizing radiation-induced Mdm2 phosphorylation at Ser429 and Ser386 (lanes 2 and 3 versus lanes 5 and 6 in pS429 and pS386 panels, respectively). Interestingly, ionizing radiation-induced phosphorylation of H2AX (Figure 6g, pSer139), a well-known ATM substrate involved in recruitment of DNA repair proteins, and p53 (Figure 6f, pSer15) was not significantly affected in *SMG7*^*−/−*^ cells compared with wild-type cells. To further examine whether SMG7 is sufficient to promote Mdm2 phosphorylation, we carried out rescue experiments by expressing SMG7 via lentiviral infection of *SMG7*^*−/−*^ cells. As shown in the Supplementary Figure S5D and F, expression of SMG7 restored DNA damage-induced Mdm2 Ser395 phosphorylation (panel E) as well as p53 stabilization (panel F), although the levels of exogenous SMG7 was only 27% of the endogenous levels (panel D). Thus, our data demonstrate that SMG7 is critical for ATM phosphorylation of Mdm2, and also suggests that SMG7 stabilizes p53 by promoting ATM-mediated Mdm2 phosphorylation upon DNA damage.

## Discussion

Here we report the identification of a previously unknown p53-binding protein SMG7, and our present data demonstrate that SMG7 has a critical role in regulation of p53-mediated response to DNA damage. Inactivation of SMG7 by gene knockout abrogates robust p53 stabilization and activation and the ability of p53 to block cell cycle progression after DNA damage; however, it appears to not significantly impact the basal steady-state level of p53 and cell growth under normal condition. Intriguingly, SMG7 physically interacts with Mdm2 in a p53-independent manner and inhibition of Mdm2 restores p53 stability in damaged *SMG7* knockout cells. Furthermore, our finding that SMG7 is necessary for ionizing radiation-induced phosphorylation of Mdm2 at multiple ATM sites provides an important molecular mechanism for SMG7-mediated p53 stabilization during DNA damage response. Therefore, our present study clearly establishes for the first time the nonsense mRNA decay factor SMG7 as a critical component of the ATM-Mdm2-p53 pathway in response to genotoxic stress.

Stabilization of p53 is critical for its role in regulation of cellular response to DNA damage and necessitates effective inhibition of Mdm2-mediated ubiquitination and degradation of p53. It has been suggested that inhibition of the Mdm2-p53 interaction by DNA damage has a role in p53 stabilization[6], which is consistent with the observations that DNA damage-induced post-translational modifications such as phosphorylation and acetylation may dissociate p53 from Mdm2 [23, 25, 57]. Previous studies also demonstrate that disruption of Mdm2-p53 binding by a small-molecule inhibitor is indeed sufficient to stabilize and activate p53 [50]. However, mass spectrometric analysis of the p53 protein complex indicates that there are significant amounts of Mdm2 bound to p53 in irradiated cells (Supplementary Figure S1B). Furthermore, our data show that, although the interaction between Mdm2 and p53 is similarly reduced in both wild-type and *SMG7*^*−/−*^ cells after DNA damage, p53 is still readily degraded by Mdm2 in the absence of SMG7 (Supplementary Figure S5A and Figure 6b). Thus, these results suggest that DNA damage-induced inhibition of Mdm2-p53 binding is insufficient for p53 stabilization.

Other molecular mechanisms that regulate p53 stability upon DNA damage have also been proposed. For example, it was shown that the Mdm2 level is transiently reduced because of accelerated degradation shortly after DNA damage, which leads to p53 stabilization [58–60]. In our present study, we did observe that Mdm2 appears to be degraded more rapidly after ionizing radiation, but knockout of *SMG7* did not show obvious effect on Mdm2 levels or its degradation before and shortly after irradiation (Figure 3e, 1 h following irradiation), suggesting that accelerated degradation of Mdm2 at the initial stage of DNA damage alone may not be sufficient for p53 stabilization. Several other studies show that ATM phosphorylation of Mdm2 is critical for p53 stabilization through inhibition of the E3 ligase activity of Mdm2 [33, 34]. Importantly, *in vivo* studies using mice bearing altered *Mdm2* alleles demonstrate that lack of Mdm2 phosphorylation at a single residue (serine 395) has a dramatic impact on p53 stabilization and activation following DNA damage [35]. Our finding that ionizing radiation-induced ATM phosphorylation of Mdm2 at Ser395 as well as two other sites Ser429 and Ser386 is abrogated in *SMG7*^*−/−*^ cells (Figure 6e) is significant in that it reveals a unique role for SMG7 in p53 stabilization.

Intriguingly, our data show that ATM can phosphorylate Mdm2 in the absence of SMG7 *in vitro* and ATM-mediated phosphorylation of p53 and H2AX is largely unaffected in *SMG7*^*−/−*^ cells (Supplementary Figure S5B and Figure 6f and g). These results suggest that SMG7 may not be essential for the overall ATM activity but has a specific role in regulation of Mdm2 phosphorylation by ATM *in vivo*. Although the precise mechanism by which SMG7 promotes Mdm2 phosphorylation remains to be elucidated, SMG7 may function as an adaptor protein that facilitates ATM phosphorylation of Mdm2 after DNA damage. Indeed, SMG7 contains an N-terminal 14-3-3-like domain and C-terminal low complex region (Figure 2a), both of which are involved in mediation of protein–protein interactions, and has been implicated as an adaptor in the nonsense-mediated mRNA decay pathway [43, 61]. Furthermore, as SMG7 physically interacts with the C-terminal region of Mdm2 (Figure 5e), which is in close proximity to the ATM phosphorylation sites including Ser368/395/429, it is conceivable that SMG7 binding may result in conformational change for Mdm2 to be an optimal ATM substrate. Previous study shows that the Wip1 phosphatase dephosphorylates Mdm2 Ser395 after DNA damage [62]; therefore, it is possible that SMG7 binding to Mdm2 may inhibit Wip1-mediated dephosphorylation.

Although our present study demonstrates that SMG7 is essential for p53 stabilization following DNA damage by regulating Mdm2 phosphorylation, it is possible that SMG7 may also regulate p53 through additional mechanisms. For example, Mdmx, an Mdm2-related protein, has a crucial role in inhibition of p53 function [7]. It has been shown that DNA damage induces phosphorylation of Mdmx at multiple C-terminal serine residues, and these modifications significantly contribute to p53 stabilization and activation [63–67]. Thus, it will be important to investigate whether SMG7 also regulates phosphorylation of Mdmx under DNA damage condition. One intriguing finding in our present study is that the interaction between endogenous SMG7 and p53 is robustly induced under various DNA damage conditions (Figure 2h and Supplementary Figure S2C). Given that both *p53* and *SMG7* homologs are conserved between mammals and ‘lower’ eukaryotic organisms such as *C. elegans* [41, 68], it will be interesting to know whether the inducible interaction between SMG7 and p53 is an evolutionarily conserved response to DNA damage? In addition, whether and how the DNA damage-inducible SMG7 binding regulates p53 stabilization and/or activation?

## Materials and Methods

### Cell culture and transfections

HCT116 cells (wild type and *p53*^*−/−*^) were obtained from Dr Bert Vogelstein, and HCT116 *p53*^*+/FH*^ and *SMG7*^*−/−*^ cell lines were generated in the present study. HCT116 and its derivative cell lines were all maintained in McCoy's 5A Medium (Cellgro, Manassas, VA, USA) supplemented with 10% fetal bovine serum. U2OS, H1299, 293 and 293FT cells were maintained in DMEM (Cellgro) supplemented with 10% fetal bovine serum. The stable cell lines were established by transfecting U2OS cells with the plasmids pCin4-Flag-HA-SMG7, followed by selection with 0.4 mg ml^−1^ G418 (GIBCO, Grand Island, NY, USA). Transfections with plasmid DNA were performed using Lipofectamine 2000 (Invitrogen, Carlsbad, CA, USA) according to the manufacturer’s protocol.

### Plasmids and antibodies

To construct pCin4-Flag/HA-SMG7, the human SMG7-coding sequence was amplified using PCR, cloned into TOPO TA cloning vector (Invitrogen) and subcloned in a frame downstream from Flag/HA epitope in the pCin4 vector. pCin4-Flag-p53 S378A/D/E, pCin4-Flag/HA-Mdm2 S395A and pGEX-2T-Mdm2 S395A plasmids were obtained by mutagenesis using the QuickChange Site-Directed Mutagenesis Kit (Stratagene, La Jolla, CA, USA) according to the manufacturer’s protocol. The presence of the mutations in the plasmids was confirmed by DNA sequencing. To construct lentiviral pGIPZ-Flag/HA-SMG7 vector, pGIPZ control short hairpin RNA plasmid was digested by *Mlu*I and *Xho*I to remove the short hairpin RNA sequence, followed by insertion of the *Nru*I–*Not*I fragment from pCin4-Flag/HA-SMG7 into the modified pGIPZ vector at *Not*I and blunted *Xba*I sites. The resultant plasmid expresses SMG7-IRES-puromycin cassette, which allows for selection of puromycin-resistant cells stably expressing SMG7. Other plasmids used in the experiments include the pCin4-Flag-p53, pCin4-HA-SMG7, pCMV-Mdm2, pCin4-Flag/HA-Mdm2 and pGEX-2T-Mdm2.

Anti-p21 (C-19, sc-397), anti-p53 (DO-1, sc-126), anti-p53 (FL-393, sc-6243), anti-p53 (1801) and anti-rabbit secondary antibodies were purchased from Santa Cruz Biotechnology Inc. (Dallas, TX, USA); mouse anti-γH2AX antibody from Upstate (Lake Placid, NY, USA); anti-Mdm2 antibody (4B11) from Calbiochem (San Diego, CA, USA); anti-SMG7 (A302-170A) antibody from Bethyl Laboratories Inc. (Montgomery, TX, USA); anti-pS15-p53 (9284), anti-ATM (D2E2, 2873) and anti-actin (4967) antibodies from Cell Signaling Technology (Beverly, MA, USA); anti-pS395-Mdm2 (PA5-13008) from Thermo Scientific Pierce (Rockford, IL, USA); anti-mouse secondary antibody (NA931) from GE (Little Chalfont, UK); anti-mouse TrueBlot secondary antibody (1-8817-31) from Rockland Inc (Limerick, PA, USA). Anti-pS386-Mdm2 and anti-pS429-Mdm2 antibodies were generous gifts from Dr Jiandong Chen.

### Purification of p53-associated proteins from HCT116 p53^+/FH^ cells

Freshly grown wild-type and *p53*^*+/FH*^ HCT116 cells were treated with 10 Gy of ionizing radiation and harvested in cold phosphate-buffered saline (PBS) 4 h later. The cell pellets were suspended in 20 times volume of ice-cold BC100 buffer (20 mM Tris-HCl, pH 7.9, 100 mM NaCl, 10% glycerol, 0.2 mM EDTA and 0.5% Triton X-100) with fresh proteinase inhibitor cocktail and incubated on ice for 1 h with several times of brief vortex. After centrifugation at 15 000 r.p.m. for 30 min at 4 °C, the supernatants were cleared by passing through 0.45-μm syringe filters and the final cell extracts were subjected to IP with anti-Flag antibody-conjugated M2 agarose beads (Sigma). The bound polypeptides eluted with the Flag peptide were further affinity-purified with anti-HA antibody-conjugated agarose, and the final elutes from the HA beads with HA peptides were resolved on an 8% SDS-PAGE gel for silver staining or were subjected to mass spectrometric analysis (by MS Bioworks LLC).

### GST pulldown

The *in vitro* translated SMG7 proteins or purified SMG7 proteins, as indicated, were incubated with 10 μl GST beads bound with 2 μg of GST or GST-fusion proteins in binding buffer BC100 in the presence of 1 μg μl^−1^ of BSA on a rotator overnight at 4 °C. The GST beads were washed five times with binding buffer and the bound proteins were eluted by boiling in SDS sample, resolved on an 8% SDS-PAGE and analyzed with western blot analysis using anti-SMG7 antibody.

### Immunoprecipitation and western blot analyses

To immunoprecipitate the Flag-tagged or Flag-HA-tagged proteins, cells were lysed in the BC100 lysis buffer with fresh proteinase inhibitor cocktail. The cell extracts were immunoprecipitated with M2 agarose beads (Sigma) and the Flag peptide elutes were resolved using SDS-PAGE and detected with antibodies as indicated. To detect the endogenous protein interaction between SMG7 and p53, or between SMG7 and Mdm2, cells were lysed in BC100 buffer with fresh proteinase inhibitor cocktail. The whole-cell extracts were immunoprecipitated with the anti-p53 (1801), anti-Mdm2 (4B11) or mouse IgG (sc-2025, Santa Cruz Biotechnology) antibody at 4 °C overnight. Protein A/G Agarose beads (#16-157, Millipore, Billerica, MA, USA) were added and incubated at 4 °C for 2 h. After the beads were washed stringently, the bound proteins were eluted by boiling in SDS sample buffer and detected with western blot analysis using anti-SMG7, anti-Mdm2 and anti-p53 antibodies. To detect phosphorylation of endogenous Mdm2, cells pretreated with 10 μM MG132 for 4 h were exposed to 10 Gy of ionizing radiation, harvested and lysed in BC100 with 0.5% SDS, proteinase inhibitor and phosphatase inhibitor (Sigma). After dilution of SDS to 0.1%, total Mdm2 proteins from cell extracts were immunoprecipitated with anti-Mdm2 (4B11) antibody and were analyzed with western blot analysis using phosphorylation-specific antibodies.

### *In vitro* kinase assay

GST-Mdm2 wild-type or S395A proteins (100 ng) prepared from bacterial were incubated without or with purified ATM proteins purified from transfected 293 cells in the kinase reaction buffer (20 mM HEPES buffer, pH 7.5, 50 mM NaCl, 10 mM MgCl_2_, 1 mM dithiothreitol, 10 mM MnCl_2_ and 5 mM ATP) for 1 h at 30 °C. The samples were then boiled and resolved on 6% SDS-PAGE gel and subjected to western blot analysis using anti-pSer395-Mdm2, anti-Mdm2 (4B11) and anti-ATM (D2E2) antibodies.

### Quantitative PCR

Total RNA was extracted from control, irradiated or Doxorubicin-treated HCT116 *SMG7*^*+/+*^ and *SMG7*^*−/−*^ cells using the TRIzoI reagent (15596018, Ambion, Austin, TX, USA) according to the manufacturer’s manual. After reverse transcription, the quantitative PCR was performed in triplicate with SYBR Green Master Mix (4309155, Applied Biosystems, Carlsbad, CA, USA) and the StepOnePlus Real-Time PCR System (Applied Biosystems) with the following PCR conditions: 10 min at 95 °C followed by 40 cycles of 95 °C for 15 s and 60 °C for 1 min. Primers used for PCR are as follows: Actin-beta (5′-CCAACCGCGAGAAGATGACC-3′ and 5′-CGTTGGCACAGCCTGGATAGCAACG-3′); *p21* (5′-CCATGTGGACCTGTCACTGTCTT-3′ and 5′-CGGCCTCTTGGAGAAGATCAGCCG-3′); *Mdm2* (5′-ATGAAAGCCTGGCTCTGTGTGT-3′ and 5′-CGGATTACACCAGCATCAAGATCCG-3′).

### Cycloheximide chase assay

Cells were untreated or exposed to 10 Gy of ionizing radiation and cycloheximide (50 mg ml^−1^ prepared in methanol) was then added for 1 h after irradiation to the culture medium at a final concentration of 50 μg ml^−1^. Cells were harvested at indicated time points and p53 in the cell extracts were examined with western blot analysis using anti-p53 antibody (DO1).

### Detection of endogenous ubiquitinated p53

Cells untreated or treated as indicated were washed with ice-cold PBS twice and lysed in BC100 buffer containing 1% SDS, proteinase inhibitors and 10 mM iodoacetamide, immediately followed by incubation at 100 °C for 5 min. The total lystes were briefly sonicated and examined by western blot analysis using anti-p53 antibody (DO1).

### Generation of HCT116 p53^+/FH^ and SMG7^*−/−*^ cell lines

The AAV-FH-p53 targeting vector was constructed based on the Flag-p53 epitope-tagging vector (a generous gift from Dr Waldman and Dr Kim) [40]. Briefly, the Flag-p53 vector DNA was used as a template in two separate PCR reactions containing primer pair A (5′-CCCGATTACGCTGAGGAGCCGCAGTCAGATC-3′ and 5′-GGAGTACTCACCCCAACAGCTG-3′) or B (5′-GGAGTGGCCAACTCCATCAC-3′ and 5′-CACGTCATAAGGGTACTTATCGTCGTCATCCTTGTAATCC-3′). The PCR fragments from A and B were digested with *Sac*I and *Age*I, respectively, and were cloned into the *Age*I and *Sac*I sites of the Flag-p53 epitope-tagging vector through three-way ligation. The presence of Flag-HA tags was confirmed using DNA sequencing. The AAV viral stocks were produced by transfection of the AAV-FH-p53 vector DNA together with pAAV-RC and pHELPER (from Stratagene) into 293FT cells using Lipofectamine 2000. Cells were harvested 48 h after transfection and lysed in PBS via three cycles of freeze/thaw in 37 °C water bath. Cleared lysates (viral stocks) were added to HCT116 cell cultures and incubated for 24 h. Cells were then split into 96-well plates and selected in the presence of 0.4 mg ml^−1^ G418 for 10 days. Individual G418-resistant clones were expanded and genotyped using PCR amplification of the genomic DNA with a pair of primers (sequences available upon request) specific for the p53-targeting locus. PCR-derived DNA fragments from clones with targeted integration of the AAV-FH-p53 vector were sequenced to confirm the insertion of the Flag-HA tag into the p53 translation start site. Correct clones were then infected with Ad-Cre viruses (from Vector Biolabs, Malvern, PA, USA) to facilitate the removal of the neomycin selection cassette. Twenty-four hours after infection, cells were serially diluted into 96-well plates and maintained in G418-free media for 2 weeks. Individual clones were expanded and tested for G418 sensitivity. Several G418-sensitive clones were examined for the expression of FH-p53 by western blot analysis and one clone was chosen for further studies.

To construct the *SMG7* knockout vector AAV-SMG7, we cloned the left and right homology arms into the pAAV-SEPT-Acceptor vector. Briefly, the left- and right-arm DNA fragments were amplified by PCR using the genomic DNA from HCT116 cells with primer pair L (5′-AAACCGGTGGGACCTTTGGCTGCCCAG-3′ and 5′-AAGAGCTCAGTCCTGCAGAGCCTGCCTG-3′) and R (5′-AAACATATGTAAGTATTACCTATCATTTGAGAAGTCC-3′ and 5′-AACTCGAGCATTTCTGATGACATCTTATCC-3′). After digestion with *Age*I/*Sac*I and *Nde*I/*Xho*I, respectively, the left- and right-arm DNA fragments were assembled into the pAAV-SEPT-Acceptor vector through four-way ligation. The AAV-SMG7 viral stocks were then produced and used for infection of HCT116 cells as described above. Individual G418-resistant clones were screened by PCR using primer pair P1 (5′-CATGGAGCAATATCAGGCTATCTGTACC-3′) and P2 (5′-GCATCAGAGCAGCCGATTGTC-3′) and partial deletion of *SMG7* exon 3 was confirmed using DNA sequencing. Clones with homologous integration of AAV-SMG7 were then infected with Ad-Cre viruses to remove the neomycin selection cassette. G418-sensitive clones containing a wild-type and a knockout *SMG7* allele (designated as *SMG7*^*+/−*^) were then subjected to the second-round targeting in the same manner to delete the remaining wild-type allele. Homozygous *SMG7* knockout was verified using PCR amplification of the targeted region using primers P3 (5′-TGGGTCCAGCTGAAGTCTGG-3′) and P4 (5′-CAGGGACTTCTCAAATGATAGGTAATACTTAC-3′), which flanks exon 3. Several clones with both *SMG7* alleles deleted were obtained, two of which were used in our present studies. Semiquantitative RT-PCR of *SMG7* and *p53* was performed using Advantage 2 Polymerase Mix (639201, Clontech, Mountain View, CA, USA) according to the manufacturer’s protocol with the following primers: *SMG7* (5′-CGGTGCTCTTTCCTGTCCCTCACCG-3′ in exon 18 and 5′-TTTGGAGCTGGATGTCAGGTTT-3′ exon 19); *p53* (5′-ATGGAGGAGCCGCAGTCAGATC-3′ and 5′-TCAGTCTGAGTCAGGCCCTTCTG-3′); *actin* (5′-CCAACCGCGAGAAGATGACC-3′ and 5′-CGTTGGCACAGCCTTGGATAGCAACG-3′).

### EDU staining

Cells were untreated or treated with 10 Gy of ionizing radiation or 200 ng ml^−1^ Doxorubicin for indicated times, and EDU was added to the medium at a final concentration of 10 μM. After incubation with EDU for 1 h, cells were fixed with 4% paraformaldehyde, permeabilized by PBS with 0.5% Triton X-100, followed by EDU staining using the Click-iT EdU Imaging Kits (Invitrogen) according to the manufacturer’s instructions. Cells were then stained by 7-AAD and analyzed using flow cytometry or stained by 4,6-diamidino-2-phenylindole and visualized with a fluorescence microscope.

### Hoechst 33258 staining

Cells treated with or without 10 Gy irradiation or 200 ng ml^−1^ of Doxorubicin were collected and gently suspended in Mccoy’s 5A medium supplemented with 10% fetal bovine serum and stained with Hoechst 33258 (H1398, Invitrogen) according to the manufacturer’s procedure. Cells were then immediately visualized under fluorescence microscope, and cells with condensed chromatin or fragmented nuclei were counted as apoptotic.

### Generation of Flag/HA-SMG7-expressing lentivirus and cell infection

293FT cells were co-transfected with pGIPZ-Flag/HA-SMG7, pCMV-dR8.91 and pMD.G plasmids with a proportion of 2:1:1 using Lipofectamine 2000 (Invitrogen), followed by change with fresh medium 16 h later. Medium supernatants containing viruses were collected for 48 or 72 h after transfection and stored at −80 °C. For infection, *SMG7*^*−/−*^ HCT116 cells were incubated with the crude lentivirus-containing medium in the presence of 8 μg ml^−1^ polybrene overnight and cells stably expressing Flag/HA-SMG7 were selected with 1 μg ml^−1^ of puromycin.
