# Supplementary material for: SMG7 is a critical regulator of p53 stability and function in DNA damage stress response
Source: Cell Discov. 2016 Jan 19;2:15042–. doi: 10.1038/celldisc.2015.42 (PMC4860962; doi:10.1038/celldisc.2015.42)
Supplement: Supplementary Figure S1 [file celldisc201542-s1.pdf]

Supplementary information, Figure S1

A

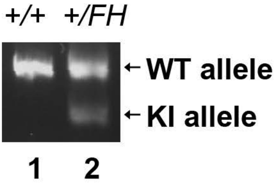

B

p53-associated protein	MW (kDa)	SpC	SAF
Mdm2	55	203	3.69
PRKDC	469	1002	2.14
SMG7	127	211	1.66
CBP	265	367	1.38
53BP1	214	208	0.97
PARC	281	268	0.95
HDAC1	55	52	0.95
MTA2	75	55	0.73
HDAC2	55	39	0.71
Cullin 7	191	102	0.53
Mdmx	55	28	0.51
USP7	128	29	0.23
ATR	301	68	0.23
Sirt1	82	15	0.18

Supplementary information, Figure S1 (related to Figure 1) List of the representative p53-interacting proteins identified from HCT116 *p53*$^{+/FH}$ knockin cells

(A) PCR genotyping analysis of genomic DNA from HCT116 *p53*$^{+/+}$ and *p53*$^{+/FH}$ knockin cells. The upper band indicates the WT allele and the lower band indicates the KI allele.

(B) A list of representative p53-associated proteins (in a descending order of their respective SAF values) identified by mass spectrometry analysis of the purified p53-containing protein complex. SpC (spectral count) is defined as the total number of spectra identified for a protein in mass spectrometry and SAF (spectral abundance factor) is defined as SpC divided by the molecular weight of the protein.
