# Supplementary material for: SMG7 is a critical regulator of p53 stability and function in DNA damage stress response
Source: Cell Discov. 2016 Jan 19;2:15042–. doi: 10.1038/celldisc.2015.42 (PMC4860962; doi:10.1038/celldisc.2015.42)
Supplement: Supplementary Figure S2 [file celldisc201542-s2.pdf]

Supplementary information, Figure S2

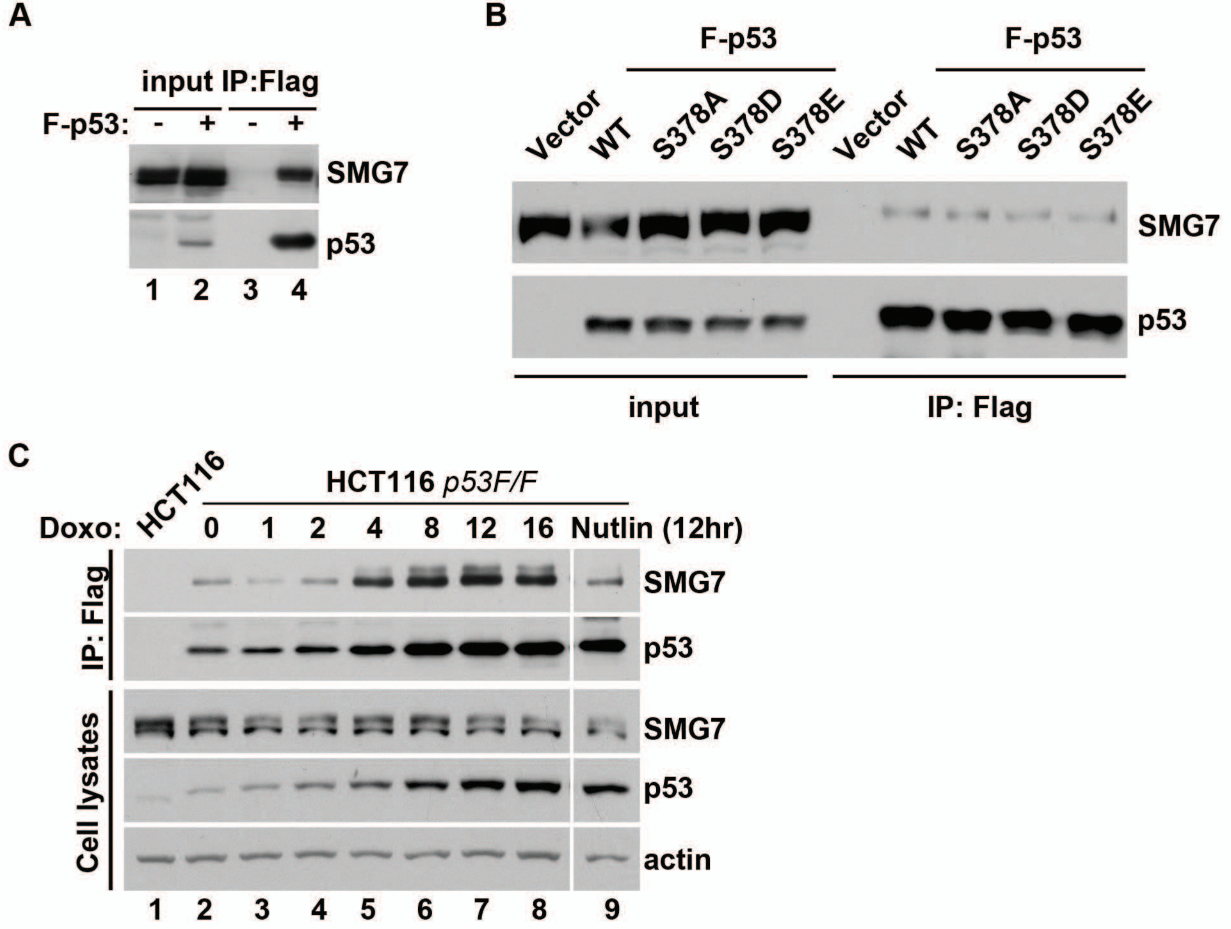

Supplementary information, Figure S2 (related to Figure 2) Interaction between SMG7 and p53

(A) H1299 cells were transfected with plasmid DNA expressing Flag-p53 and/or SMG7, as indicated. The cell extracts and the α-Flag immunoprecipitates were analyzed by western blot using antibodies against SMG7 and p53 (DO-1).

(B) H1299 cells were transfected with plasmid DNA expressing Flag-p53 wild-type, S378A, S378D, S378E and/or SMG7, as indicated. The cell extracts and the anti-Flag immunoprecipitates were analyzed by western blot using antibodies against SMG7 and p53 (DO-1).

(C) HCT116 p53+/+ and p53F/F cells treated without (lanes 1 and 2) or with 200 ng/ml of Doxorubicin (lanes 3-8), or with 10 uM Nutlin (lane 9) were harvested at the indicated time points. The cell lysates were subjected to anti-Flag immunoprecipitation, and the cell extracts (lower panel) and the immunoprecipitates (upper panel) were analyzed by western blot using anti-SMG7, anti-p53 (DO-1), and anti-actin antibodies.
