# Supplementary material for: SMG7 is a critical regulator of p53 stability and function in DNA damage stress response
Source: Cell Discov. 2016 Jan 19;2:15042–. doi: 10.1038/celldisc.2015.42 (PMC4860962; doi:10.1038/celldisc.2015.42)
Supplement: Supplementary Figure S3 [file celldisc201542-s3.pdf]

Supplementary information, Figure S3

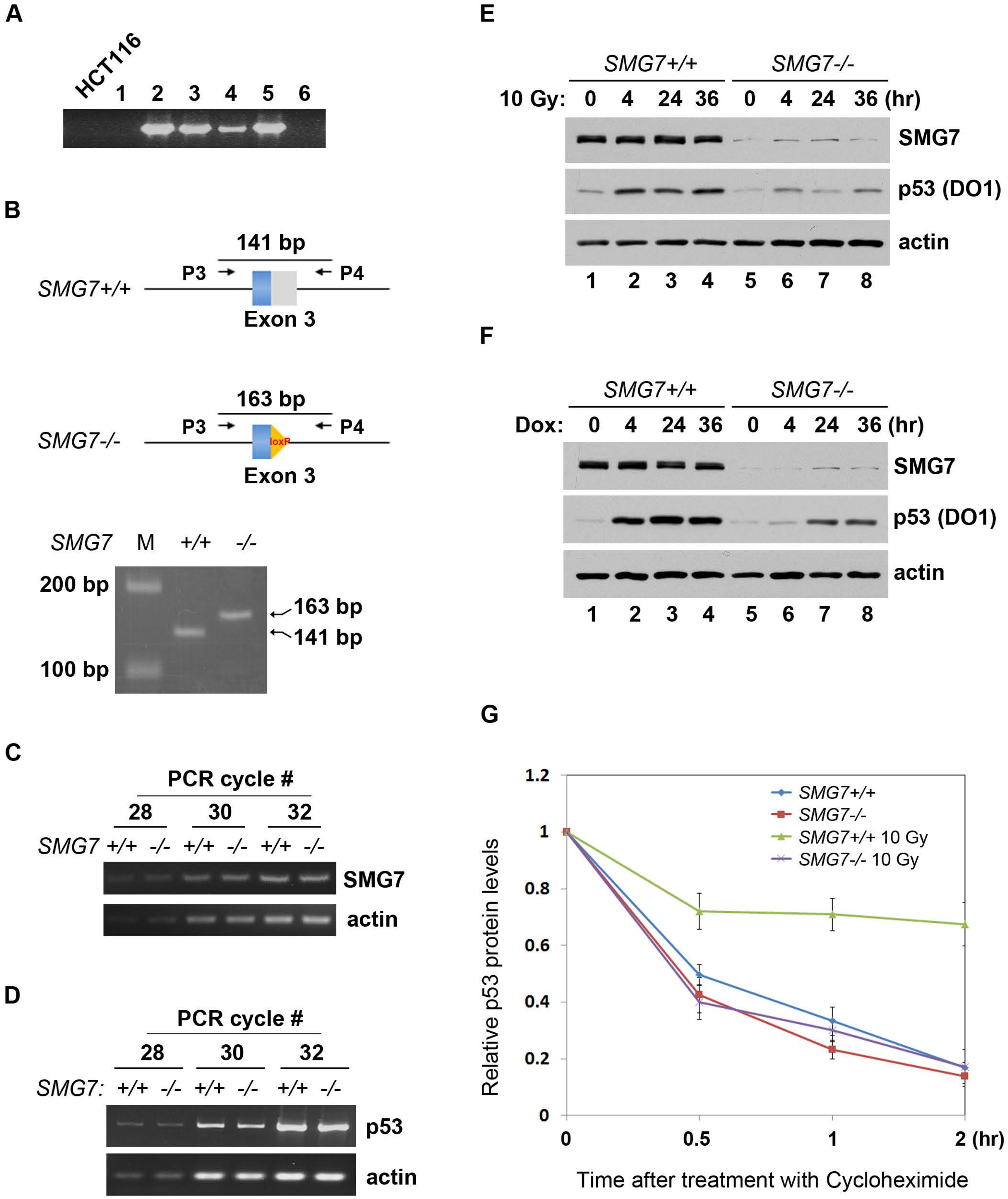

Supplementary information, Figure S3 (related to Figure 3) Identification/characterization of *SMG7* knockout cells and quantitation of p53 levels in *SMG7* knockout cells. (See details of PCR conditions including primer sequences in **Materials and Methods f**or **(A) – (D)**.)

(A) PCR genotyping analysis of genomic DNA from HCT116 $SMG7^{+/+}$ and $SMG7^{+/IRES\text{-}neo}$ clones.

(B) PCR genotyping analysis of genomic DNA from HCT116 $SMG7^{+/+}$ and $SMG7^{-/-}$ cells.

(C) *SMG7* and *Actin* mRNA levels from HCT116 $SMG7^{+/+}$ and $SMG7^{-/-}$ cells are determined by semi-quantitative RT-PCR analysis.

(D) *p53* and *Actin* mRNA levels from HCT116 $SMG7^{+/+}$ and $SMG7^{-/-}$ cells are determined by semi-quantitative RT-PCR analysis.

(E) Cell extracts from control and irradiated (10 Gy, 4, 24, and 36 hours) were analyzed by western blot with α-SMG7, α-p53 and α-actin antibodies.

(F) Cell extracts from control and Doxorubicin-treated (200 ng/ml, 4, 24, and 36 hours) were analyzed by western blot with α-SMG7, α-p53 and α-actin antibodies.

(G) Quantitation of p53 protein levels in HCT116 $SMG7^{+/+}$ and $SMG7^{-/-}$ cells treated as in **Figure 3E** using ImageJ. Data represented three independent experiments and values are mean ± S.E..
