# Supplementary material for: SMG7 is a critical regulator of p53 stability and function in DNA damage stress response
Source: Cell Discov. 2016 Jan 19;2:15042–. doi: 10.1038/celldisc.2015.42 (PMC4860962; doi:10.1038/celldisc.2015.42)
Supplement: Supplementary Figure S4 [file celldisc201542-s4.pdf]

Supplementary information, Figure S4

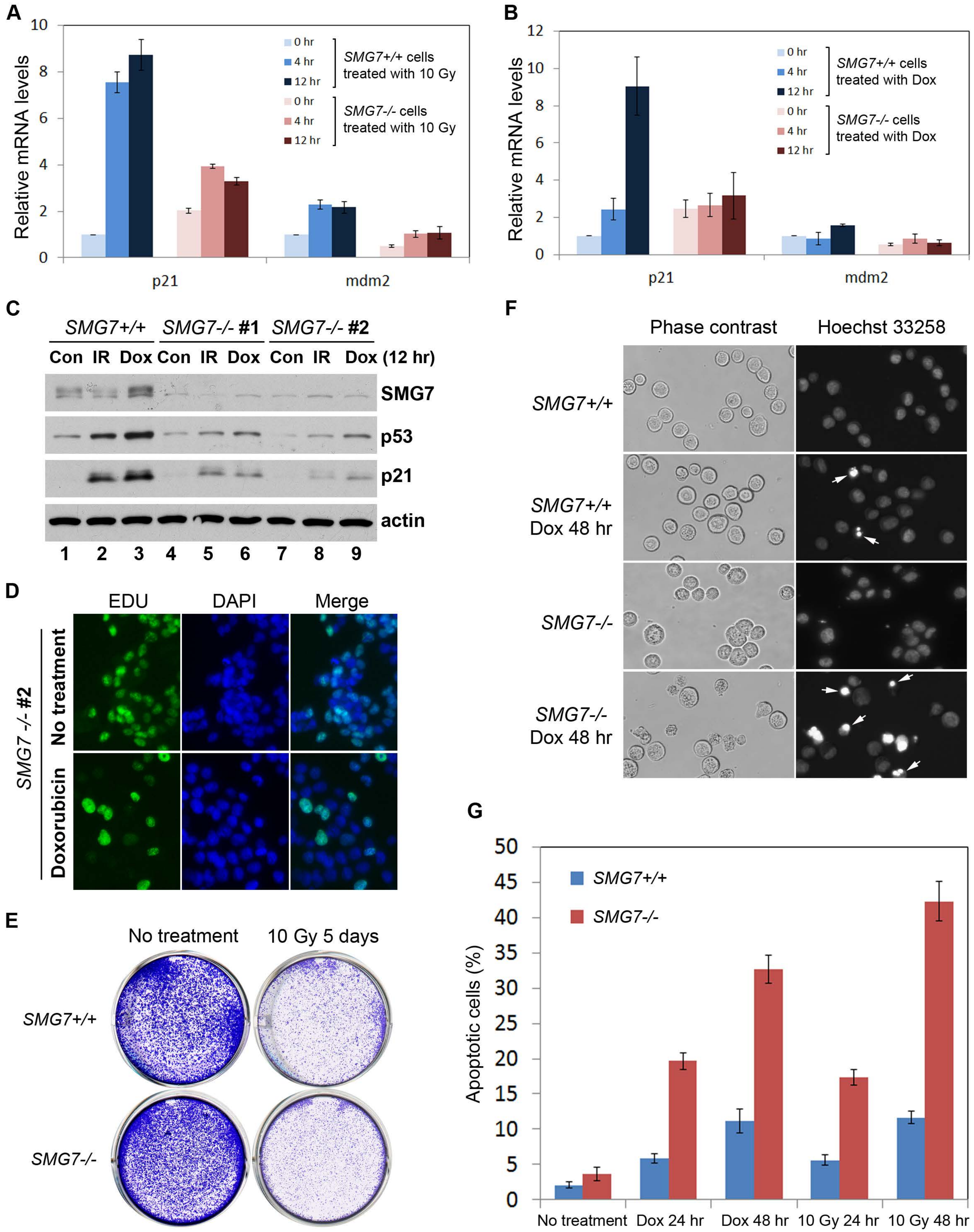

Supplementary information, Figure S4 (related to Figure 4) SMG7 is required for p53 stabilization and activation following DNA damage

(A) Quantitative real-time PCR analysis of *p21* and *Mdm2* mRNA levels in HCT116 $SMG7^{+/+}$ and $SMG7^{-/-}$ cells either untreated or irradiated (10 Gy, 4 and 12 hours).

(B) Quantitative real-time PCR analysis of *p21* and *Mdm2* mRNA levels in HCT116 $SMG7^{+/+}$ and $SMG7^{-/-}$ cells treated with or without 200 ng/ml Doxorubicin (4 and 12 hours).

(C) HCT116 $SMG7^{+/+}$ and $SMG7^{-/-}$ (two individual clones) cells were treated without (lanes 1, 4, and 7) or with 10 Gy of IR (lanes 2, 5, and 8), or with 200 ng/ml of Doxorubicin (lanes 3, 6, and 9), and harvested 12 hours after treatment. The total cell extracts were analyzed by western blot using the antibodies against SMG7, p53 (DO-1), p21, and actin.

(D) HCT116 $SMG7^{-/-}$ cells (a second clone) were treated without or with 200 ng/ml of Doxorubicin for 16 hour and 10 uM of EDU was added for 1 hour before harvest. The cells were fixed and processed for EDU (green) and DAPI (blue) staining, followed by microscopic analysis.

(E) $SMG7^{+/+}$ and $SMG7^{-/-}$ cells were untreated or irradiated with 10 Gy. After growth for 5 days, cells were washed with PBS and stained with crystal violet.

(F) Untreated and Doxorubicin-treated (200 ng/ml, 48 hours) $SMG7^{+/+}$ and $SMG7^{-/-}$ cells were harvested and stained with Hoechst 33258, followed by fluorescence microscopy. Apoptotic cells were identified by bright nuclear staining with condensed or fragmented nuclei, some of which are indicated by white arrows.

(G) $SMG7^{+/+}$ and $SMG7^{-/-}$ cells were either untreated or treated with irradiated (10 Gy, 24 and 48 hours) or Doxorubicin (200 ng/ml, 24 and 48 hours). Cells were stained with Hoechst 33258 and analyzed as in **F**. The percentage of apoptotic cells were shown in the graph and the data represented three independent experiments. Values are mean ± S.E..
