# Supplementary material for: SMG7 is a critical regulator of p53 stability and function in DNA damage stress response
Source: Cell Discov. 2016 Jan 19;2:15042–. doi: 10.1038/celldisc.2015.42 (PMC4860962; doi:10.1038/celldisc.2015.42)
Supplement: Supplementary Figure S5 [file celldisc201542-s5.pdf]

Supplementary information, Figure S5

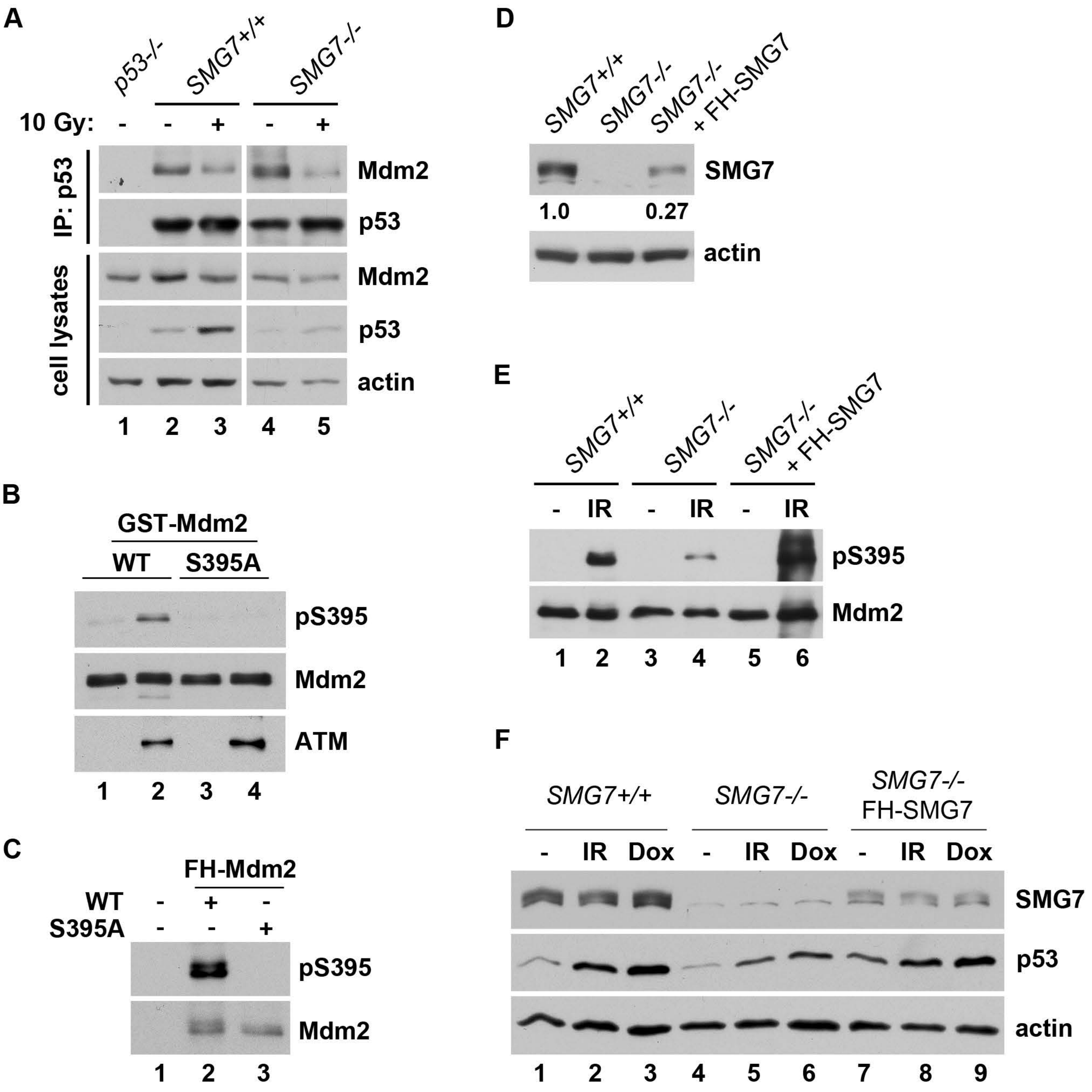

Supplementary information, Figure S5 (related to Figure 6) In vivo binding of p53 with Mdm2 in *SMG7* knockout cells, characterization of the p-S395-Mdm2 antibody and rescue of Mdm2 phosphorylation by expression of SMG7

(**A**) Wild-type, *p53*$^{-/-}$, and *SMG7*$^{-/-}$ HCT116 cells were treated without (lanes 1-2, and 4) or with 10 Gy of IR (lanes 3 and 5) and harvested after 1 hr. The cell extracts were subjected to immunoprecipitation with α-p53 antibody (1801), and the total cell extracts (lower panel) and the immunoprecipitates (upper panel) were analyzed by western blot using antibodies against Mdm2, p53 (DO-1) and actin.

(B) In vitro phosphorylation of Mdm2 S395 by ATM. Wild-type and S395A mutant GST-Mdm2 proteins were incubated without or with ATM immunoprecipitated from transfected 293 cells. The reaction products were analyzed by western blot with anti-Mdm2 S395 phosphorylation specific antibody, anti-Mdm2 antibody (4B-11), and anti-ATM antibody (D2E2).

(C) 293 cells were transfected with plasmid DNA expressing Flag-HA-tagged Mdm2 and treated with 10 Gy of IR, and cells were harvested after 1 hour. The cell extracts were subjected to anti Flag immunoprecipitation and the immunoprecipitates were analyzed by western blot with α-Mdm2 S395 phosphorylation-specific antibody and anti-Mdm2 antibody (4B-11).

(D) Western blot analysis of the total cell extracts with α-SMG7 and α-actin antibodies. The SMG7 protein bands in lanes 1 and 3 were quantitated using ImageJ and the numbers below the band (1 and 0.27, respectively) indicate the relative levels of SMG7.

(E) Cells were treated with or without IR (10 Gy, 1 hour). The Mdm2 proteins were immunoprecipitated using the α-Mdm2 (4B11) antibody were analyzed by western blot with α-pS395-Mdm2 and rabbit α-Mdm2 antibodies.

(F) Cell extracts from control, irradiated (10 Gy, 4 hours) and Doxorubicin-treated (200 ng/ml, 6 hours) were analyzed by western blot with α-SMG7, α-p53 and α-actin antibodies.
